# Enteric nervous system disease in neuronopathic lysosomal storage disorders

**DOI:** 10.4103/NRR.NRR-D-25-00448

**Published:** 2025-08-13

**Authors:** Ewa A. Ziółkowska, Robert O. Heuckeroth, Jonathan D. Cooper

**Affiliations:** Department of Pediatrics, Washington University in St. Louis, School of Medicine, St. Louis, MO, USA; Children’s Hospital of Philadelphia Research Institute and Perelman School of Medicine at the University of Pennsylvania, Philadelphia, PA, USA; Department of Genetics, Washington University in St. Louis, School of Medicine, St. Louis, MO, USA; Department of Neurology, Washington University in St. Louis, School of Medicine, St. Louis, MO, USA

**Lysosomal storage disorders and their impact upon the central nervous system:** Lysosomal storage disorders (LSDs) are a group of over 70 rare inherited metabolic disorders (Platt et al., 2018). They are caused by dysfunction of lysosomes, organelles that contain enzymes responsible for digesting macromolecules. In functional lysosomes, these enzymes break down complex substrates, and the resulting fragments are recycled. Individual LSDs are caused by mutations in genes that encode lysosomal enzymes or other proteins crucial for lysosome function (Platt et al., 2018). These mutations cause lysosomal dysfunction and intralysosomal accumulation of undigested substances. This accumulation of “storage material” does not necessarily directly damage cells, but other poorly understood consequences of lysosomal dysfunction impair cell health over time. The vast majority of LSDs are inherited in an autosomal recessive or X-linked recessive manner (Platt et al., 2018). Although individually rare, the overall prevalence of LSDs is estimated at 1 in 5000 live births. Symptoms of LSDs are diverse and progressive, significantly affecting quality of life of patients, and ultimately proving fatal (Platt et al., 2018). Approximately two thirds of LSDs have severe effects on the central nervous system (CNS) characterized by progressive neurodegeneration (Platt et al., 2018). Research into these “neuronopathic” LSDs has primarily focused on the CNS, but there is increasing evidence that these LSDs also damage the peripheral nervous system, including recently demonstrated damage to the enteric nervous system (ENS).

**Critical role of the enteric nervous system in regulating bowel function:** The ENS, sometimes referred to as the “second brain,” is a vast network of neurons and glia that controls bowel motility, epithelial function, blood flow, and immune cell activities in response to local stimuli (Sharkey and Mawe, 2023). To perform these roles there are ~20 neuron types and ~8 types of enteric glia, with about as many neurons in the bowel as in the spinal cord (Sharkey and Mawe, 2023). These neurons and glia are distributed along the entire length of the bowel in two interconnected networks: myenteric plexus ganglia between bowel smooth muscle layers and submucosal plexus ganglia between circular muscle and epithelium (Sharkey and Mawe, 2023). Not surprisingly, ENS dysfunction causes diverse gastrointestinal (GI) symptoms, including constipation, diarrhea, abdominal distension, and vomiting as manifestations of dysmotility or epithelial dysfunction, and ENS loss may predispose to bowel inflammation (Sharkey and Mawe, 2023).

**Gastrointestinal symptoms in lysosomal storage disorders — a historical perspective:** In LSDs, bowel symptoms are common according to anecdotal reports. These symptoms not only worsen with age and impair life quality but may ultimately prove life-threatening. Nevertheless, very little is formally published about these GI symptoms in LSDs, which have often been attributed to CNS dysfunction, impaired mobility, poor hydration, or medication side effects. Remarkably, the accumulation of storage material in enteric neurons was noted in several LSDs over 50 years ago. Before molecular diagnoses were possible, these disorders were diagnosed via rectal biopsies to visualize this storage in enteric neurons (Brett and Lake, 1975). This ENS phenotype was also reported at autopsy and in a variety of mouse models, but links to GI symptoms were not pursued.

**New evidence for enteric nervous system involvement in lysosomal storage disorders:** Only recently has ENS disease, including enteric neuron loss been described in several LSDs. First, when CNS-directed gene therapy failed to effectively treat a cat model of Sandhoff disease, life-limiting systemic manifestations, including in the ENS, were recognized as a possible explanation for treatment failure (Johnson et al., 2023). More recently, our groups reported profound enteric neuron loss and bowel dysmotility in mouse models of CLN1 and CLN2 disease, forms of neuronal ceroid lipofuscinosis (NCL, or Batten disease) (Ziółkowska et al., 2025). In these disorders, the ENS appears to form normally but then undergoes progressive neurodegeneration (Ziółkowska et al., 2025). These ENS defects could be prevented by gene therapy, significantly extending survival of treated mice. Interestingly, enteric neuron loss was not evenly distributed, but occurred in patches, a phenotype also evident in human autopsy bowel (Ziółkowska et al., 2025). Such findings provide a new perspective on how GI symptoms may arise in LSDs, and how these disorders may ultimately be treated. To improve outcomes, we need to more fully define the impact of LSDs on the ENS and determine how ENS degeneration contributes to disease progression. Further mechanistic research may facilitate new targeted therapies and enable more comprehensive and effective treatment for both the CNS and peripheral nervous system in LSDs. As discussed below, these novel findings raise a number of fundamental questions about how the bowel is affected in CNS neurodegenerative disorders.

**Importance of the “gut–brain axis” in health and disease:** While the impact of the CNS on bowel function is well-known, recent studies highlight the bidirectional nature of this influence, often described as the “gut–brain axis” (GBA) (Cryan et al., 2019). This term covers complex reciprocal communications between the CNS and the bowel, that impact the function of the nervous system, mucosal immune cells, endocrine cells, and gut microbiota (Cryan et al., 2019). Evidence from several CNS disorders suggests dysregulation of the GBA may not only contribute to these disorders, but that ENS dysfunction may also drive CNS disease (Hajjeh et al., 2025). While causality is yet to be fully established, these examples provide new perspectives on the pathogenesis of CNS disease, and also novel potential strategies for their treatment.

**The enteric nervous system as a driver of neurodegeneration in the central nervous system — Parkinson’s disease:** The occurrence of early gastrointestinal symptoms in Parkinson’s disease (PD) has long been recognized, being mentioned prominently in the original description of PD. Parkinson even suggested that a “disordered state of the stomach and bowels may induce a morbid action” in the brain. Recent evidence has substantiated this suggestion, describing the spread of characteristic alpha-synuclein deposits from ENS neurons to the brain via the vagus nerve (reviewed in Tan et al., 2022). The potential for altered intestinal permeability and inflammation within the bowel and the influence of gut microbial imbalance (dysbiosis) and gut microbiota–derived metabolites have all been considered contributors to PD (Tan et al., 2022). These issues have been investigated in experimental models of PD and in large cohorts of PD patients (Tan et al., 2022), to examine the influence of dietary modifications, prebiotics, probiotics, and fecal microbiota transplants on disease progression. These interventions have not always produced consistent results but remain an area of continued interest.

**Altered neuroimmune reactions — evidence from multiple sclerosis:** Another neurologic disorder for which the contributory role of GBA is actively considered is multiple sclerosis (MS), a disorder characterized by inflammatory demyelination within the CNS. Many studies documented dysbiosis and associated increased intestinal permeability in people with MS and MS mouse models (Parodi et al., 2021). “Leaky gut” may allow bacterial products and metabolites to move across the bowel lining, disrupting normal tissue homeostasis, and provoking local and systemic immune responses, including in the CNS.

**A role for dysbiosis?** In addition to PD and MS, contributory roles for a perturbed GBA and dysbiosis have been proposed for depression, anxiety, autism, and disorders of appetite control (Cryan et al., 2019). A wealth of information regarding bowel phenotypes and dysbiosis has been generated in each disorder, but it remains unclear to what degree dysbiosis is a pathological trigger or simply a consequence of the disease. Nevertheless, the drugs commonly used to treat these disorders also produce a complex range of effects on the microbiome. Mechanistically establishing cause-and-effect in such studies of the GBA is not straightforward. For NCLs, for example, gut dysbiosis has been described in mouse models, but it is not known if dysbiosis contributes to, or is caused by, NCL disease progression.

**Research challenges to understanding the enteric nervous system contribution to rare diseases:** Research on the ENS in rare neurodegenerative diseases faces numerous challenges. Most fundamental is that these disorders are predominantly considered to affect the CNS. As a result, the bowel is rarely collected at autopsy and there is a limited availability of such material for research. Because enteric neurons cluster into ganglia that are unevenly distributed within the bowel, studies based on thin tissue sections may not adequately sample ENS neuropathology unless many sections are evaluated or whole mount imaging strategies are employed. Methods to study bowel function, including imaging and electrophysiology also face technical challenges, but techniques are rapidly improving, especially for model systems. Although bowel-specific methodologies and the understanding of ENS neurobiology have lagged behind CNS research, recent years have seen vast progress.

**Key directions for future research:** Our discovery of progressive neurodegeneration within the ENS in LSDs previously considered to be primarily CNS disorders (Ziółkowska et al., 2025), opens up significant new opportunities for understanding disease mechanisms and devising effective therapies. In these LSDs, enteric neuron loss occurs in localized patches where no neurons are present, while surrounding areas appear less affected. This could suggest that neuropathology in one cell accelerates neurodegeneration in nearby cells perhaps via abnormal neuron-to-neuron interactions such as the spread of misfolded alpha-synuclein in PD. Alternatively, if interactions between enteric neurons and glia are compromised, impaired glial support may render neurons more vulnerable, as appears to occur in the CNS in LSDs. Similarly, neuropathology could be driven by altered neuroimmune interactions involving different classes of lymphocytes or bowel macrophages to promote a proinflammatory environment as occurs in MS. It is not yet clear whether any specific enteric neuron subtype is affected more than others, but this is under investigation.

**Gaining insights into pathophysiology in the enteric nervous system:** Cellular interactions and the selective vulnerability of different neuron or glial subtypes need to be determined in the ENS. Depending on the neuron types most vulnerable, ENS neurodegeneration might alter the bowel epithelial barrier or immune cell function in addition to effects on bowel motility. These changes could cause dysbiosis, increasing levels of pro-inflammatory cytokines or microbe-derived small molecules that in turn accelerate ENS or CNS disease progression. In addition, lysosomal dysfunction may directly negatively impact other bowel cell types impairing their normal function. Studies to address how different bowel cell types are affected by LSDs and of cellular autonomy of lysosomal enzyme deficiency are expected to yield valuable data. Understanding these events will be important for devising targeted means to treat these newly identified LSD enteric phenotypes.

**Therapeutic implications:** In addition to gene therapy or enzyme replacement therapy for LSDs, bowel-targeted strategies might include prebiotics, probiotics, selective microbiome editing, or engineering microbial communities to produce beneficial small molecules. For some CNS disorders (migraine, seizures, and PD), vagal nerve stimulation improves outcomes, but the impact of neuromodulation in LSDs is yet to be investigated. As disease mechanisms are clarified, druggable targets within the bowel may be identified, facilitating repurposing of US Food and Drug Administration-approved pharmaceuticals. Strategies for targeting the ENS by gene therapy should also be improved to increase the transduction of ENS cells and reduce off-target effects of viral vectors (such as hepatocellular carcinoma). Based on previous gene therapy experience in the CNS, there is likely a threshold of vector transduction, enzyme expression, and activity that needs to be reached to improve outcomes. Lysosomal enzymes released by one cell are taken up by adjacent cells where they can correct their lysosomal enzyme deficiency, a process termed “cross-correction.” This means not all cells need direct viral transduction to receive the benefits of gene therapy, but defining the optimal cellular targets of gene therapy could still improve outcomes. Finally, we hypothesize that optimal clinical outcomes will require targeting both CNS and ENS, a major paradigm shift for planned therapies.

**Summary and prospects:** Research on the ENS in neurodegenerative diseases, including the NCLs and other LSDs, is in its infancy, but already highlights potentially significant contributions of newly defined bowel phenotypes to disease outcomes. The ENS not only regulates gastrointestinal functions but also serves as a crucial component of the gut-brain axis, influencing the course of some CNS neurodegenerative disorders (Hajjeh et al., 2025; **Figure 1**). The pathological changes so far documented in the ENS of LSD models include widespread yet localized enteric neuron degeneration, with changes in glial cell populations, and immune cell activation that appear to differ in some ways from the better understood events that occur within the CNS. Indeed, with different cellular players and different immune privilege compared to the CNS, bowel-specific mechanisms may operate. Nevertheless, the ENS remains insufficiently studied in the context of rare diseases, primarily due to failing to consider that this key regulator of bowel function may also affected in these diseases. Understanding the underlying disease mechanisms within the bowel will undoubtedly help in the development of more effective therapies, including those that target both bowel and CNS.

**Figure 1:**
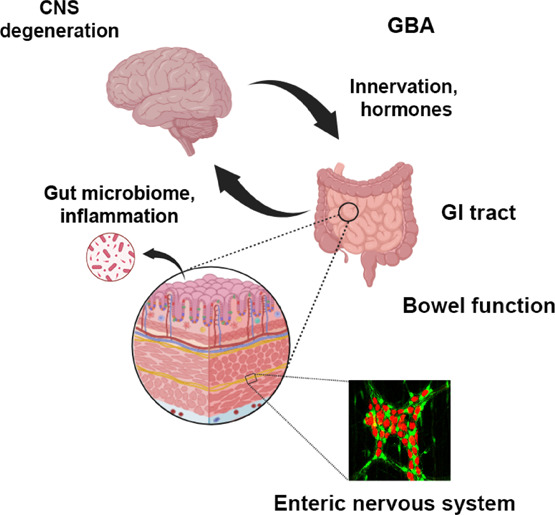
Schematic representation of potential links between CNS degeneration, bowel function, the gut microbiome, and enteric nervous system via the GBA. Unpublished data. Created with BioRender.com and Adobe Photoshop. CNS: Central nervous system; GBA: gut–brain axis; GI: gastrointestinal.

In conclusion, the ENS represents a promising, yet underexplored, area in the study of neurodegenerative diseases. Incorporating ENS research into NCL pathogenesis studies and developing therapeutic strategies that consider ENS roles could significantly enhance our understanding of these diseases and improve patients’ quality of life.

**Additional file:** *Open peer review report 1.*

OPEN PEER REVIEW REPORT 1

## References

[R1] Brett EM, Lake BD (1975). Reassessment of rectal approach to neuropathology in childhood: review of 307 biopsies over 11 years. Arch Dis Child.

[R2] Cryan JF (2019). The microbiota-gut-brain axis. Physiol Rev.

[R3] Hajjeh O, Rajab I, Bdair M, Saife S, Zahran A, Nazzal I, AbuZahra MI, Jallad H, Abukhalil MM, Hallak M, Al-Said OS, Al-Braik R, Sawaftah Z, Milhem F, Almur O, Saife S, Aburemaileh M, Abuhilal A (2025). Enteric nervous system dysfunction as a driver of central nervous system disorders: The Forgotten brain in neurological disease. Neuroscience.

[R4] Johnson AK, McCurdy VJ, Gray-Edwards HL, Maguire AS, Cochran JN, Gross AL, Skinner HE, Randle AN, Shirley JL, Brunson BL, Bradbury AM, Leroy SG, Hwang M, Rockwell HE, Cox NR, Baker HJ, Seyfried TN, Sena-Esteves M, Martin DR (2023). Life-limiting peripheral organ dysfunction in feline sandhoff disease emerges after effective CNS gene therapy. Ann Neurol.

[R5] Mole SE, Anderson G, Band HA, Berkovic SF, Cooper JD, Kleine Holthaus SM, McKay TR, Medina DL, Rahim AA, Schulz A, Smith AJ (2019). Clinical challenges and future therapeutic approaches for neuronal ceroid lipofuscinosis. Lancet Neurol.

[R6] Parodi B, Kerlero de Rosbo N (2021). The gut-brain axis in multiple sclerosis. Is its dysfunction a pathological trigger or a consequence of the disease?. Front Immunol.

[R7] Platt FM, d’Azzo A, Davidson BL, Neufeld EF, Tifft CJ (2018). Lysosomal storage diseases. Nat Rev Dis Primers.

[R8] Sharkey KA, Mawe GM (2023). The enteric nervous system. Physiol Rev.

[R9] Tan AH, Lim SY, Lang AE (2022). The microbiome-gut-brain axis in Parkinson disease-from basic research to the clinic. Nat Rev Neurol.

[R10] Ziółkowska EA (2025). Gene therapy prevents bowel dysmotility, enteric neuron degeneration and extends survival in lysosomal storage disorder mouse models. Sci Transl Med.

