# Supplementary material for: Enteric nervous system disease in neuronopathic lysosomal storage disorders
Source: Neural Regen Res. 2025 Aug 13;21(8):3537–8. doi: 10.4103/NRR.NRR-D-25-00448 (PMC13452649; doi:10.4103/NRR.NRR-D-25-00448)
Supplement: Supplementary file 1 [file NRR-21-3537_Suppl1.pdf]

OPEN PEER REVIEW REPORT 1

Name of journal: Neural Regeneration Research
Manuscript NO: NRR-D-25-00448
Title: Exploring enteric nervous system disease in neuronopathic lysosomal storage disorders
Reviewer's Name: Júlio César Claudino Dos Santos
Reviewer's country: Brazil

COMMENTS TO AUTHORS

This is a thought-provoking and well-referenced Perspective article. It introduces an important and underappreciated aspect of LSD pathogenesis by examining ENS involvement. The integration of historical findings with current gene therapy insights is particularly strong. I recommend adding section headings for better navigation, elaborating slightly on the mechanisms of selective ENS vulnerability, and considering the inclusion of a schematic figure summarizing key concepts and proposed pathways. These minor improvements will enhance clarity and impact.

Minor Concerns and Suggestions:

1. Structural Clarity: While the narrative is coherent, the manuscript would benefit from clearer subheadings to guide the reader through the sections (e.g., "Historical Evidence", "ENS Pathophysiology in LSDs", "Future Therapeutic Approaches").

2. Mechanistic Details: Although this is a perspective article, a slightly more detailed discussion of the hypothesized mechanisms driving selective ENS vulnerability (e.g., neuron-glia interactions, neuroimmune signaling) could enrich the reader's understanding.

3. Illustrative Figure: A schematic summarizing the proposed interactions between the CNS, ENS, gut microbiota, and potential therapeutic targets would enhance the visual impact of the article.

4. Terminology Clarification: Terms like "patchy degeneration" and "cross-correction" are used appropriately, but a brief definition or example could assist readers less familiar with gene therapy or ENS pathology.

This is a valuable and thought-provoking Perspective article that opens new avenues in the understanding of LSDs and neurogastroenterology. I recommend acceptance with minor revisions as indicated above to further strengthen its clarity and impact.
